# Association between antibiotic use and pathologic response to neoadjuvant chemotherapy in breast cancer: a multicentre retrospective cohort study

**DOI:** 10.1016/j.breast.2026.104833

**Published:** 2026-06-11

**Authors:** M. Zalabardo, I. Fernández, A. Girona, Á. González, J. Blanco, Ó. Campos, A. Sánchez-Muñoz, J.L. Onieva, J.M. Jerez, J. Pascual, M.J. Bermejo, B. Jiménez, A. Márquez, B.I. Pajares, M. Domínguez, A. Godoy-Ortiz, T. Díaz, I. Zarcos, F. Carabantes, E. Villar, N. Ribelles, E. Alba

**Affiliations:** a Department of Medicine, University of Málaga, Málaga, Spain; b Department of Medical Oncology, Virgen de la Victoria University Hospital, Málaga, Spain; c B-01 Group, Biomedical Research Institute of Málaga (IBIMA), Málaga, Spain; d Applied Bioinformatics Unit, Center for Research and Advanced Cancer Therapies (CITAC), Málaga, Spain; e Department of Languages and Computer Science, University of Málaga, Málaga, Spain; f Biomedical Research Networking Center in Oncology (CIBERONC), Madrid, Spain; g Department of Medical Oncology, Regional University Hospital of Málaga, Málaga, Spain; h Department of Medical Oncology, Costa del Sol University Hospital, Marbella, Spain

**Keywords:** Breast cancer, Neoadjuvant chemotherapy, Antibiotic exposure, Residual cancer burden, Pathologic response

## Abstract

**Background:**

Antibiotics (ATBs) are frequently prescribed during neoadjuvant chemotherapy (NACT) for localized breast cancer, but their impact on pathologic response is uncertain**.**

**Patients and methods:**

We conducted a retrospective multicenter cohort study of women treated with NACT between January 2009 and January 2024 at three university hospitals in Spain. ATB exposure was ≥1 systemic course within 30 days before NACT or during NACT until surgery. Pathologic response was assessed using the Residual Cancer Burden (RCB) index; endpoints were optimal response (RCB-0/I) and pathologic complete response (pCR; RCB-0). Associations were examined using multivariable logistic regression adjusting for clinical, pathologic, and treatment factors, including relative dose intensity (RDI) and ECOG performance status.

**Results:**

Among 1316 patients, 516 (39.2%) received antibiotics. RCB-0/I was less frequent in exposed vs unexposed patients (36.4% vs 48.6%; *P* < 0.001); RDI ≥85% was maintained in 83.9% of exposed patients. After adjustment, ATB exposure was associated with lower odds of RCB-0/I (OR 0.56, 95% CI 0.43–0.72; *P* < 0.001). In subtype-stratified models, ATB exposure was associated with lower odds of RCB-0/I across luminal, HER2-positive, and triple-negative disease (adjusted OR range, 0.54–0.56; *P* < 0.05), without significant ATB × subtype interaction. ATB exposure was associated with lower pCR odds (OR, 0.75; 95% CI, 0.57–0.98; *P* = 0.03), despite nonsignificant unadjusted rates (27.7% vs 32.4%; *P* = 0.08).

**Conclusions:**

Antibiotic exposure shortly before or during NACT was associated with a reduced likelihood of achieving RCB-0/I and pCR after accounting for treatment delivery. These findings support antibiotic stewardship, reinforce that clinically indicated antibiotics remain essential, and require prospective validation.

## Highlights

• Antibiotic exposure occurred in 39.2% (516/1316) of breast cancer NACT patients.
• Optimal RCB-0/I was lower with antibiotics: 36.4% vs 48.6% (*P* < 0.001).
• Antibiotic exposure had lower RDI/ECOG-adjusted RCB-0/I odds (OR 0.56; *P* < 0.001).
• Antibiotic exposure had lower adjusted pCR odds (OR 0.75; *P* = 0.03).
• Antibiotics lowered RCB-0/I across subtypes (ORs 0.54–0.56); interaction *P* = 0.81.

## 1 Introduction

Breast cancer (BC) remains a health challenge, ranking as the second most common cancer in incidence and first in cancer-related deaths among women in 2022 [1]. It is a biologically heterogeneous disease shaped by genomic, cellular, and host-related factors that drive distinct clinical behaviours and treatment responses. Tumors with similar phenotypes, defined by traditional clinical-pathologic profiles [2], may exhibit divergent outcomes, reflecting underlying genomic complexity and patient-specific biological factors [3]. This has prompted a shift toward personalized strategies incorporating both tumor-intrinsic and host-related features [4,5].

Neoadjuvant chemotherapy (NACT) is a standard approach for locally advanced or aggressive tumors, offering outcomes comparable to those of adjuvant therapy [6] while enabling an early assessment of efficacy through pathologic response [7,8]. Pathologic complete response (pCR), defined as the absence of invasive cancer in the breast and axillary nodes (ypT0/is, ypN0), is strongly associated with favorable prognosis at the individual-patient level, particularly in triple-negative, HER2-positive, and high-grade luminal tumors [[9], [10], [11]].

Antibiotics (ATBs) are frequently used during NACT, with population-based estimates suggesting that approximately one in five patients require ATBs during treatment and that around 10% are hospitalized for infection-related complications [12]. Comparable ATB use has been observed in selected neoadjuvant BC cohorts [13]. However, ATBs can disrupt the gut microbiota, impairing metabolic and immune functions essential for host homeostasis [14,15].

Human microbiota, especially the gut ecosystem, is essential to immunomodulation, epithelial barrier integrity, and xenobiotic metabolism [[16], [17], [18], [19]]. Growing evidence implicates it as a key regulator of antitumor immunity, treatment efficacy, and prognosis [20,21]. Most microbiota–cancer evidence derives from colorectal and immunogenic tumors treated with immune checkpoint inhibitors (ICI), where ATBs are linked to worse outcomes [[22], [23], [24]]. Preliminary data suggest a harmful effect of ATBs on treatment outcomes in BC, though evidence remains limited [25].

Nonetheless, the impact of ATB exposure during NACT remains poorly defined, particularly in non-immunotherapy settings, which remain largely unexplored despite their clinical relevance [[24], [25], [26]]. While preclinical and translational studies suggest that ATB-induced dysbiosis may modulate antitumor immunity and drug response [14,16,20,26], clinical evidence in BC is scarce. To address this gap, we conducted a retrospective multicenter study to evaluate whether ATB exposure was associated with pathologic response to NACT in patients with early-stage BC.

## 2 Methods

### 2.1 Study design

We conducted an observational, retrospective, multicenter cohort study including patients with early BC who received NACT between January 2009 and January 2024 at three university hospitals in Spain (Hospital Virgen de la Victoria, Hospital Regional Universitario de Málaga, and Hospital Costa del Sol). All patients had complete follow-up through surgery. The objective was to evaluate the association between ATB use and pathologic response to NACT, measured using the Residual Cancer Burden (RCB) index [27].

### 2.2 Antibiotic exposure

ATB exposure was defined as receiving at least one course of systemic ATBs within 30 days before the start of NACT or at any time during NACT until surgery. Patients were classified as exposed or unexposed based on this time window. This threshold was based on prior studies linking recent ATB use to worse oncologic outcomes, particularly when administered in the month before systemic therapy [[28], [29], [30]]. Similar windows have been applied in chemotherapy and ICI cohorts [25,30], with additional detrimental effects reported in BC when ATBs are administered during NACT [25]. To account for the potential relevance of timing, exposure was categorized as pre-NACT only, during NACT only, or both. No systematic prophylactic ATB strategy was included in institutional NACT protocols during the study period.

### 2.3 Population and data source

A total of 1870 women with localized BC who initiated NACT were identified through the Galen database, a shared electronic health record system across the participating hospitals [31]. Clinical records were reviewed to collect demographic, clinical, pathologic, and treatment-related variables. Information on ATB exposure was retrieved from hospital electronic medical records, including inpatient prescription and administration records, oncology day-unit charts, and the shared outpatient prescribing module used in primary and specialized care. Key variables were verified across available electronic sources. Core details on data source governance, study registration, and data accessibility are provided in the Supplementary Methods (Section 1.1).

Patients lost to follow-up before surgery (*N* = 194), those treated with ICI-based regimens (*N* = 28), or those with incomplete data due to undocumented treatment dosing or ATB exposure (*N* = 332) were excluded, yielding a final cohort of 1316 patients (Supplementary Fig. S1). “Undocumented” dosing or ATB exposure was defined as the absence of reliable information on at least one key element (drug names, cumulative dose, delays, number of courses, confirmation of dispensing in the outpatient pharmacy or clinical indication) across all electronic sources. These exclusions aimed to minimize exposure misclassification.

This study followed the STROBE reporting guidelines for cohort studies [32]. Data were anonymized and managed in accordance with the Declaration of Helsinki, with approval from the local Ethics Committee, which waived the requirement for informed consent.

### 2.4 Variables and measures

The primary outcome was optimal pathologic response, assessed using the RCB index, calculated according to the Symmans method [27]. The RCB index is a continuous, class-based measure of residual disease that has been reproducibly validated as a strong prognostic marker; in multiple cohorts, patients with RCB-0 and RCB-I have similarly excellent long-term outcomes, whereas prognosis deteriorates progressively for RCB-II and RCB-III [27,[33], [34], [35]]. For analysis, we applied the standard RCB classes: RCB-0 (pCR), RCB-I (minimal residual disease), RCB-II (moderate residual disease), and RCB-III (extensive residual disease) [27]. Accordingly, we prespecified the primary endpoint as the composite outcome RCB-0/I vs RCB-II/III, reflecting this prognostic distinction**.** The secondary endpoint was RCB-0 vs RCB-I/II/III.

Recorded variables included clinical (age, menopausal status, ECOG performance status, clinical tumor category [cT], clinical nodal status [cN], clinical stage [TNM], age-adjusted Charlson Comorbidity Index), pathologic (grade, Ki-67 index, histology, immunohistochemical [IHC] subtype), and treatment-related variables (NACT regimen, anthracycline use, relative dose intensity [RDI], planned and received cycles, omitted cycles and NACT prolongation >14 days) and ATB-related variables (number of courses, timing of exposure, route of administration, ATB class, and clinical indication).

Baseline comorbidity was summarized using the Charlson Comorbidity Index and prespecified clinical comorbidity domains: diabetes/metabolic, cardiovascular, pulmonary, renal, hepatic, neurological, autoimmune/inflammatory and psychiatric disorders. Detailed domain definitions are provided in the Supplementary Methods. (Section 1.2). A grouped baseline comorbidity variable was generated according to the number of affected domains (0, 1, or ≥2).

Tumors were classified into 5 IHC subtypes: luminal A (hormone receptor–positive [HR+]/HER2-, Ki-67 < 20%), luminal B (HR+/HER2-, Ki-67 ≥ 20%), HER2-positive/HR-positive (HR+/HER2+), HER2-positive/HR-negative (HR−/HER2+), and triple-negative (HR-/HER2-) [36]. For analysis, tumors were grouped into luminal (A and B), HER2-positive (both HR+ and HR-) and triple-negative based on biological and therapeutic similarity to enhance clinical interpretability.

Due to the observational design, NACT regimens varied over time. Most patients received anthracycline/cyclophosphamide–taxane combinations, with or without anti-HER2 agents, and a minority received alternative regimens. For RDI analyses, we focused on anthracyclines, cyclophosphamide, and taxanes in patients treated with standard regimens with modifiable dosing and complete treatment records (Supplementary Methods Section 1.3). For each cytotoxic drug (anthracyclines, cyclophosphamide, and taxanes), RDI was calculated as the ratio of delivered dose intensity (DDI) — total dose received (mg/m^2^) divided by actual treatment duration (days) — to the protocol-defined standard dose intensity (SDI) for the planned regimen, multiplied by 100 and expressed as a percentage. For combination regimens, global RDI was calculated as the arithmetic mean of the individual drug RDIs. Treatment duration was defined from NACT initiation to the last planned cycle; for omitted cycles, a dose of 0 and the standard cycle length were imputed, following previously described methods [37]. Global RDI<85% was considered reduced intensity, a threshold associated with lower efficacy and worse survival in early BC [[38], [39], [40], [41]]. RDI was analyzed both as a continuous variable and as a categorical variable (<85% vs ≥ 85%).

### 2.5 Statistical analysis

Categorical variables were summarized as N (%) and compared using χ^2^ or Fisher exact tests, as appropriate. Continuous variables were summarized as median (IQR) or mean (SD) and compared using Wilcoxon rank-sum tests. Two-sided P values < 0.05 were considered statistically significant. Missing covariate values were imputed using the modal category.

Multivariable logistic regression was used to assess the adjusted association between ATB exposure and favorable pathologic response, defined as RCB-0/I vs RCB-II/III and RCB-0 vs RCB-I/II/III. Candidate covariates were selected a priori on clinical grounds and included clinical, pathologic, treatment-related, and ATB-related variables. Collinearity was assessed using the variance inflation factor (VIF), with VIF≥5 indicating problematic multicollinearity. Potential confounding was explored by comparing the crude ATB OR with models adjusted individually for each prespecified non–ATB-related covariate; a >10% change in the ATB OR was considered relevant. ATB burden descriptors, including number of courses, route, and indication, were considered components of the exposure construct and were not entered as independent confounders in the primary models.

Subsequently, multivariable models were built using a bidirectional stepwise selection procedure based on the Akaike Information Criterion. RDI was included a priori to account for its potential role as a confounder or mediator of the association between infection/ATB use and pathologic response and to estimate the ATB–response association after accounting for measured treatment delivery. ECOG performance status was also forced into the final models to account for baseline functional status. Models were prespecified for the overall cohort and repeated within each IHC subtype. Sensitivity analyses compared models using categorical vs continuous RDI, models restricted to patients with preserved global RDI (≥85%), and models incorporating the age-adjusted Charlson Comorbidity Index to assess the robustness of ATB–response associations to treatment delivery and baseline frailty/comorbidity. Formal interaction testing was performed by adding an ATB exposure × grouped IHC subtype interaction term to the overall model and comparing nested models using a likelihood-ratio χ^2^ test.

ORs and 95% CIs were estimated for each favorable pathologic response endpoint (RCB-0/I and RCB-0), using reference categories with the highest theoretical likelihood of response to yield conservative and clinically interpretable estimates of the ATB–response association. The final models were evaluated using the Hosmer–Lemeshow goodness-of-fit test to assess calibration and a likelihood ratio test comparing the full model with a reduced model excluding ATB exposure to determine its incremental statistical contribution. All analyses were performed using R, version 4.3.1 (R Foundation for Statistical Computing). Data were analyzed between January 2024 and July 2024. The statistical analysis plan is provided in Supplementary Methods (Section 1.4).

## 3 Results

Of 1316 patients, 516 (39.2%) received ≥1 ATB course within 30 days before or during NACT; 800 (60.8%) were unexposed. The median age was 50 years (IQR, 43–59). Tumors were predominantly biologically aggressive, with high-grade disease in 54.0% and Ki-67 ≥ 20% in 82.5% of cases. Most tumors were classified as T2 (62.6%), N1 was the most frequent nodal status (45.6%), and stage II disease predominated (64.9%). Baseline ECOG was similar between ATB-exposed and unexposed patients (Table 1). Charlson Comorbidity Index, grouped comorbidity burden, and individual comorbidity domains were also generally balanced according to ATB exposure, except for psychiatric disorders, which were less frequent among ATB-exposed patients (Supplementary Table S1).

**Table 1:** Baseline characteristics overall and by antibiotic exposure.

	OVERALL (N = 1316), N (%)Para Run-on	NO ANTIBIOTIC (*N* = 800), *N* (%)	ANTIBIOTIC (*N* = 516), *N* (%)	*P*
Age range				
<50	633 (48.1)	388 (48.5)	245 (47.5)	0.62
50-70	604 (45.9)	368 (46)	236 (45.7)	
>70	79 (6.0)	44 (5.5)	35 (6.8)	
**ECOG performance status**				
0	1139 (86.4)	692 (86.5)	447 (86.6)	1
≥1	177 (13.5)	108 (13.5)	69 (13.4)	
**Menopausal status**				
Pre/perimenopausal	727 (55.2)	446 (55.8)	281 (54.5)	0.65
Postmenopausal	589 (44.8)	354 (44.2)	235 (45.5)	
**Histological type**				
Ductal	1154 (87.7)	700 (87.5)	454 (88)	0.86
Non-ductal	162 (12.3)	100 (12.5)	64 (12)	
**Histological Grade**				
1-2	604 (45.9)	378 (47.2)	226 (43.8)	0.21
3	712 (54.1)	422 (52.8)	290 (56.2)	
**Ki-67 index**				
<20%	229 (17.5)	135 (16.9)	94 (18.3)	0.84
≥20%	1082 (82.5)	662 (83.1)	420 (81.7)	
NA^a^	5	3	2	
**Tumor (cT)**				
T1	151 (11.5)	85 (10.6)	66 (12.8)	0.11
T2	824 (62.6)	503 (62.9)	321 (62.2)	
T3	232 (17.6)	153 (19.1)	79 (15.3)	
T4	109 (8.3)	59 (7.4)	50 (9.7)	
**Node (cN)**				
N0	500 (38)	314 (39.2)	186 (36)	0.59
N1	600 (45.6)	354 (44.3)	246 (47.7)	
N2	163 (12.4)	98 (12.3)	65 (12.6)	
N3	53 (4.0)	34 (4.2)	19 (3.7)	
**Stage (TNM). AJCC 8th edition**				
I	62 (4.7)	39 (4.9)	23 (4.5)	0.92
II	854 (64.9)	520 (65)	334 (64.7)	
III	400 (30.4)	241 (30.1)	159 (30.8)	
**Grouped IHC subtype**				
Luminal	468 (35.6)	285 (35.6)	183 (35.5)	1
HER2-positive	515 (39.1)	313 (39.1)	202 (39.2)	
Triple-negative	333 (25.3)	202 (25.3)	131 (25.4)	
**Detailed IHC subtype**				
Luminal A	152 (11.6)	86 (10.8)	66 (12.8)	
Luminal B	316 (24.0)	199 (24.9)	117 (22.7)	
HER2-positive/HR-positive	333 (25.3)	201 (25.1)	132 (25.6)	
HER2-positive/HR-negative	182 (13.8)	112 (14.0)	70 (13.6)	
Triple-negative	333 (25.3)	202 (25.3)	131 (25.4)	
**Neoadjuvant Chemotherapy Regimens**				
AC-DTX	321 (24.4)	201 (25.1)	120 (23.3)	0.79
AC-PTX	429 (32.6)	254 (31.8)	175 (33.9)	
CT + dHER2	307 (23.3)	189 (23.6)	118 (22.9)	
CT + sHER2	199 (15.1)	117 (14.6)	82 (15.9)	
Other regimens	60 (4.6)	39 (4.9)	21 (4.1)	

Data are *N* (%). *P* values from χ^2^ test or Fisher exact test, as appropriate. Detailed IHC subtypes are descriptive only; all inferential analyses used the 3-category grouped subtype (Luminal vs HER2-positive vs Triple-negative).

ECOG, Eastern Cooperative Oncology Group; IHC, immunohistochemical; HR, hormone receptor; NA, not available; AC-DTX, anthracycline/cyclophosphamide → docetaxel; AC-PTX, anthracycline/cyclophosphamide → paclitaxel; CT + dHER2, chemotherapy plus trastuzumab/pertuzumab; CT + sHER2, chemotherapy plus trastuzumab.

a NA indicates missing data.

Among ATB-exposed patients, 323 (62.6%) received 1 course, 153 (29.7%) received 2, and 40 (7.8%) received ≥3 courses. β-lactams were the most common class, frequently combined with quinolones; full details are shown in Supplementary Table S2. Most ATBs were administered orally (443 [85.8%]), while intravenous-only and combined oral/intravenous administration were less frequent (33 [6.4%] and 40 [7.8%], respectively). Mean ATB treatment duration was 8.4 days (SD, 4.2). ATB exposure occurred predominantly during NACT (460 [89.2%]), whereas pre-NACT-only exposure was uncommon (26 [5.0%]); 30 patients (5.8%) received ATBs both before and during NACT. Febrile neutropenia was the most frequent indication (171 [33.1% of ATB-exposed patients; 13.0% of the full cohort]), but most ATB use corresponded to other documented infections, mainly respiratory infection (135 [26.2%]), urinary tract infection (99 [19.2%]), soft-tissue infection (63 [12.2%]), dental infection (31 [6.0%]), and other less common infections (17 [3.3%]) (Supplementary Fig. S2).

NACT regimens reflected standard protocol-based choices according to tumor subtype and patient characteristics. Most patients received anthracycline-, cyclophosphamide-, and taxane-based regimens, with or without anti-HER2 agents. Treatments were grouped into five analytic categories: anthracyclines plus docetaxel (AC-DTX), anthracyclines plus paclitaxel (AC-PTX), chemotherapy with dual anti-HER2 blockade (CT + dHER2), chemotherapy with single-agent anti-HER2 therapy (CT + sHER2), and other regimens. Their distribution did not differ by ATB exposure (P = 0.78; Supplementary Table S3). Given known toxicity differences between taxanes, taxane type was also examined and was balanced between groups: docetaxel use was similar in unexposed and exposed patients (45.1% vs. 41.3%; P = 0.19). Supplementary Table S3 lists all administered regimens, and Supplementary Table S4 details their classification.

To further characterize treatment delivery, we examined global RDI and selected markers of treatment disruption. RDI was generally high across the cohort: 1149 of 1316 patients (87.3%) achieved a global RDI ≥85%, including 433 of 516 ATB-exposed patients (83.9%). Reduced global RDI (<85%) was more frequent among ATB-exposed than unexposed patients (83 of 516 [16.1%] vs 84 of 800 [10.5%]; *P* < 0.01), although the absolute difference was modest. Mean RDI remained around 90% in both groups for anthracyclines, taxanes, and cyclophosphamide, and mean global RDI was 90.2% in exposed vs 93.0% in unexposed patients (*P* < 0.01). ATB-exposed patients had slightly more omitted cycles and clinically relevant NACT prolongation >14 days, whereas the mean number of planned and received cycles was broadly similar between groups (Table 2).

**Table 2:** Relative dose intensity and treatment delivery by antibiotic exposure.

			OVERALL (*N* = 1316)	NO ANTIBIOTIC (*N* = 800)	ANTIBIOTIC (*N* = 516)	*P*
Relative dose intensity						
Chemotherapy agent						
Anthracyclines						
RDI (continuous); mean % (SD)			90.0 (24.6)	91.3 (23.6)	88.1 (26)	0.02
RDI categorical; N (%)		<85%	158 (12)	80 (10)	78 (15.1)	0.01
		≥85%	1158 (88)	720 (90)	438 (84.8)	
Cyclophosphamide						
RDI (continuous); mean % (SD)			92.7 (19.5)	93.6 (18.9)	91.3 (20.4)	<0.01
RDI categorical; N (%)		<85%	123 (9.4)	62 (7.8)	61 (11.8)	0.01
		≥85%	1193 (90.6)	738 (92.2)	455 (88.2)	
Taxanes						
RDI (continuous); mean % (SD)			90.8 (16.4)	91.9 (15.5)	88.9 (17.5)	0.03
RDI categorical; N (%)		<85%	255 (19.4)	135 (16.9)	120 (23.3)	0.02
		≥85%	1061 (80.6)	665 (83.1)	396 (76.7)	
Global RDI						
RDI (continuous); mean % (SD)			91.9 (16.4)	93.0 (15.7)	90.2 (17.3)	<0.01
RDI categorical; N (%)		<85%	167 (12.7)	84 (10.5)	83 (16.1)	<0.01
		≥85%	1149 (87.3)	716 (89.5)	433 (83.9)	
**Treatment delivery**						
Planned cycles; mean (SD)			7.85 (0.6)	7.86 (0.6)	7.84 (0.7)	0.68
Received cycles; mean (SD)			7.69 (0.9)	7.72 (0.87)	7.65 (0.95)	0.23
Patients with omitted cycles; N (%)		No	1198 (91)	739 (92.4)	459 (89)	0.04
		Yes	118 (9)	61 (7.6)	57 (11)	
Clinically relevant NACT prolongation >14 days; N (%)		No	1174 (89.2)	729 (91.1)	445 (86.2)	<0.01
		Yes	142 (10.8)	71 (8.9)	71 (13.8)	

Data are mean (standard deviation, SD) for continuous variables and No. (%) for categorical variables. P values were calculated using the Wilcoxon rank-sum test for continuous variables and the χ^2^ or Fisher exact test for categorical variables, as appropriate. Clinically relevant NACT prolongation was defined as actual NACT duration exceeding theoretical duration by more than 14 days.

RDI, relative dose intensity; NACT, neoadjuvant chemotherapy.

According to pathologic response, 402 of 1316 patients (30.5%) achieved RCB-0, 175 (13.3%) RCB-I, 457 (34.7%) RCB-II, and 282 (21.5%) RCB-III. Optimal response, defined as RCB-0/I, was significantly more frequent among patients not exposed to ATBs than among those who were exposed (389 of 800 [48.6%] vs 188 of 516 [36.4%]; *P* < 0.001). By contrast, the proportion of patients achieving pathologic complete response (RCB-0) was similar between unexposed and ATB-exposed groups (259 of 800 [32.4%] vs 143 of 516 [27.7%]; *P* = 0.08). Graphical distributions of RCB-0/I and RCB-0 by IHC subtype and ATB exposure are shown in Supplementary Figs. S3 and S4.

Subgroup analyses by grouped IHC subtype (luminal, HER2-positive, and triple-negative) revealed consistent patterns. Among HER2-positive (*N* = 515), RCB-0/I was significantly more frequent among unexposed patients: 215 of 313 (68.7%) vs 108 of 202 (53.5%) (*P* < 0.001). RCB-0 was achieved in 151 of 313 unexposed patients (48.2%) vs 87 of 202 exposed patients (43.1%) (*P* = 0.29).

In triple-negative (*N* = 333), RCB-0/I rates were 110 of 202 unexposed patients (54.5%) and 57 of 131 exposed patients (43.5%), respectively (*P* = 0.07), while RCB-0 occurred in 79 of 202 (39.1%) and 45 of 131 (34.4%) (*P* = 0.45).

Among luminal tumors (*N* = 468), RCB-0/I was significantly more frequent in the unexposed group: 64 of 285 (22.5%) vs 23 of 183 (12.6%) (*P* = 0.01). RCB-0 was observed in 29 of 285 unexposed patients (10.2%) vs 11 of 183 exposed (6.0%) (*P* = 0.16).

Two multivariable logistic regression models were constructed to evaluate the adjusted association between ATB exposure and pathologic response. In the global models, immunohistochemical subtype and chemotherapy regimen exhibited VIF values > 20, consistent with severe multicollinearity. Accordingly, chemotherapy regimen was excluded from the multivariable models of the whole cohort, while subtype was retained and regimen was explored only in subtype-stratified analyses. In a covariate-by-covariate confounding assessment, none of the prespecified non–ATB-related covariates modified the ATB OR by >10% (Supplementary Table S5).

**Fig. 1:**
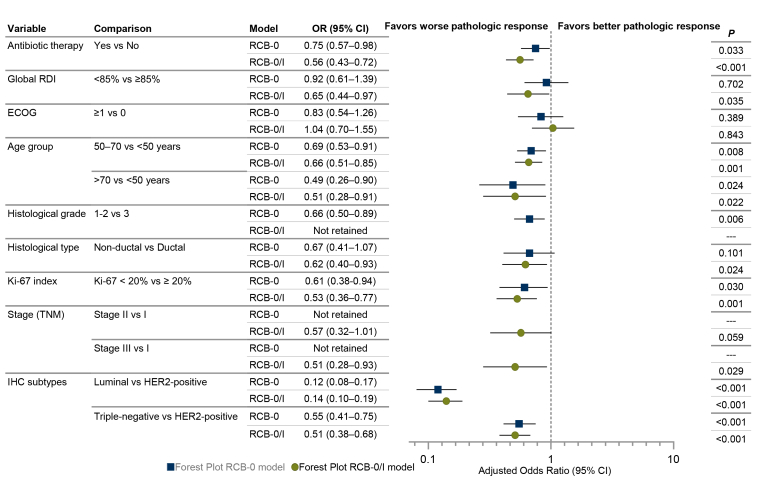
**Multivariable predictors of pathologic response in the overall cohort (RCB-0 and RCB-0/I)**. Forest plot of adjusted odds ratios (ORs) and 95% confidence intervals (CIs) from multivariable logistic regression models for pathologic complete response (RCB-0; squares) and optimal pathologic response (RCB-0/I; circles) in the overall cohort (N = 1316). ORs are shown on a logarithmic scale with OR = 1 as the null. Comparisons are shown as category vs reference; predictors not retained in each model are labeled “Not retained.” ORs <1 indicate lower odds of achieving the corresponding favorable pathologic response. RCB, residual cancer burden; OR, odds ratio; CI, confidence interval; RDI, relative dose intensity; ECOG, Eastern Cooperative Oncology Group; TNM, tumor–node–metastasis; IHC, immunohistochemical.

In the overall multivariable models, ATB exposure was associated with lower odds of achieving RCB-0/I (OR, 0.56; 95% CI, 0.43–0.72; P < 0.001). Reduced global RDI (<85%) was also associated with lower odds of RCB-0/I (OR, 0.65; 95% CI, 0.44–0.97; P = 0.035). For pCR (RCB-0), ATB exposure was also associated with lower odds of response (OR, 0.75; 95% CI, 0.57–0.98; P = 0.03), whereas global RDI was not significantly associated with pCR. ECOG performance status was not associated with either endpoint. Covariates retained in each model are shown in Fig. 1.

Both models demonstrated good calibration (Hosmer–Lemeshow *P* = 0.57 for pCR and *P* = 0.60 for RCB-0/I). Likelihood ratio tests comparing the full models with reduced models excluding ATB exposure showed that ATB use provided a statistically significant incremental contribution to model fit (*P* = 0.03 for pCR and *P* < 0.01 for RCB-0/I).

**Fig. 2:**
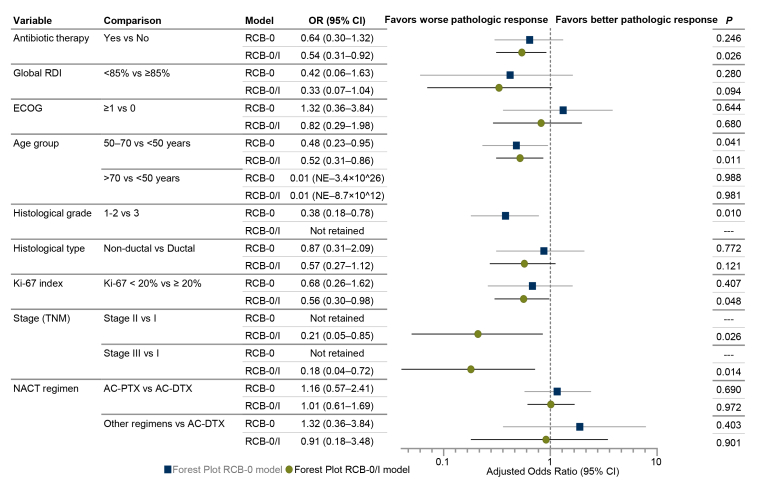
**Multivariable predictors of pathologic response in luminal breast cancer (RCB-0 and RCB-0/I)**. Forest plot of adjusted ORs (95% CIs) for pathologic complete response (RCB-0; squares) and optimal pathologic response (RCB-0/I; circles) from multivariable logistic regression models restricted to luminal tumors. ORs are displayed on a logarithmic scale with OR = 1 as the null. Comparisons are shown as category vs reference; predictors not retained in a given model are labeled “Not retained.” ORs <1 indicate lower odds of achieving the corresponding favorable pathologic response. Estimates for patients aged >70 years have very wide or non-estimable CIs and are not displayed when they extend beyond the plotting range; numerical estimates are reported in the corresponding results table. RCB, residual cancer burden; OR, odds ratio; CI, confidence interval; RDI, relative dose intensity; ECOG, Eastern Cooperative Oncology Group; NE, not estimable; TNM, tumor–node–metastasis; AC-PTX, anthracycline/cyclophosphamide → paclitaxel; AC-DTX, anthracycline/cyclophosphamide → docetaxel.

**Fig. 3:**
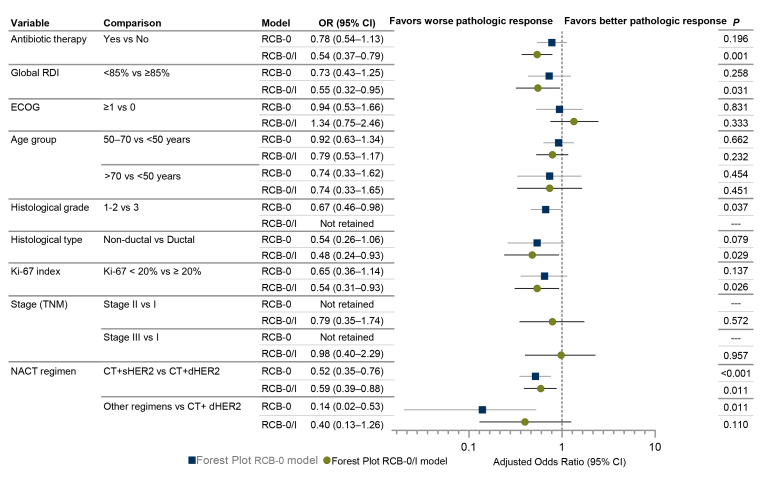
**Multivariable predictors of pathologic response in HER2-positive breast cancer (RCB-0 and RCB-0/I)**. Forest plot of adjusted ORs (95% CIs) for pathologic complete response (RCB-0; squares) and optimal pathologic response (RCB-0/I; circles) from multivariable logistic regression models restricted to HER2-positive tumors. ORs are displayed on a logarithmic scale with OR = 1 as the null. Comparisons are shown as category vs reference; predictors not retained in a given model are labeled “Not retained.” ORs <1 indicate lower odds of achieving the corresponding favorable pathologic response. HER2, human epidermal growth factor receptor 2; RCB, residual cancer burden; OR, odds ratio; CI, confidence interval; RDI, relative dose intensity; ECOG, Eastern Cooperative Oncology Group; TNM, tumor–node–metastasis; CT + sHER2, chemotherapy plus single-agent anti-HER2 therapy; CT + dHER2, chemotherapy plus dual anti-HER2 blockade.

**Fig. 4:**
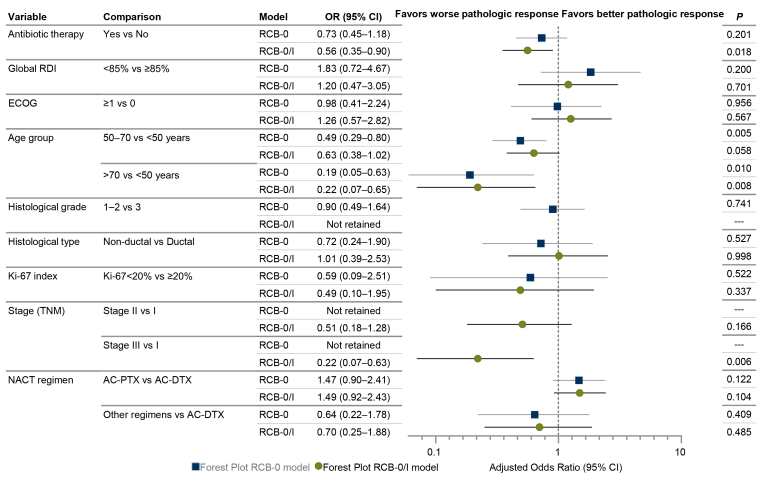
**Multivariable predictors of pathologic response in triple-negative breast cancer (RCB-0 and RCB-0/I)**. Forest plot of adjusted ORs (95% CIs) for pathologic complete response (RCB-0; squares) and optimal pathologic response (RCB-0/I; circles) from multivariable logistic regression models restricted to triple-negative tumors. ORs are displayed on a logarithmic scale with OR = 1 as the null. Comparisons are shown as category vs reference; predictors not retained in a given model are labeled “Not retained.” ORs <1 indicate lower odds of achieving the corresponding favorable pathologic response. RCB, residual cancer burden; OR, odds ratio; CI, confidence interval; RDI, relative dose intensity; ECOG, Eastern Cooperative Oncology Group; TNM, tumor–node–metastasis; AC-PTX, anthracycline/cyclophosphamide → paclitaxel; AC-DTX, anthracycline/cyclophosphamide → docetaxel.

In subtype-specific analyses, the negative association between ATB exposure and RCB-0/I was consistent across luminal (Fig. 2), HER2-positive (Fig. 3), and triple-negative tumors (Fig. 4), with adjusted ORs ranging from 0.54 to 0.56 and all *P* < 0.05. In contrast, ATB exposure was not significantly associated with pCR within individual subtypes, reflecting the more modest effect size and more limited power of subtype-restricted analyses. Formal interaction testing in the overall models did not show evidence that the association between ATB exposure and pathologic response differed significantly by grouped IHC subtype (ATB × subtype interaction *P* = 0.81 for RCB-0/I and *P* = 0.79 for RCB-0).

Sensitivity analyses supported the robustness of the main findings. Modelling global RDI as a continuous percentage yielded nearly identical estimates for ATB exposure for both RCB-0/I and RCB-0. Because age group and the age-adjusted Charlson Comorbidity Index showed collinearity, the Charlson sensitivity model was fitted by replacing age group with the age-adjusted Charlson Comorbidity Index while retaining ECOG performance status; ATB effect estimates remained virtually unchanged. In analyses restricted to patients with preserved global RDI (≥85%), ATB exposure remained associated with lower odds of RCB-0/I (OR, 0.58; 95% CI, 0.45–0.76; *P* < 0.001), whereas the association with RCB-0 was attenuated and no longer statistically significant (OR, 0.79; 95% CI, 0.59–1.05; *P* = 0.11). (Supplementary Table S6).

## 4 Discussion

NACT is a cornerstone of curative-intent treatment in early-stage BC, and pathologic response provides an early readout of treatment efficacy. Because ATBs are frequently prescribed during NACT, clarifying whether recent exposure is associated with pathologic response is clinically relevant and may inform the broader question of how supportive-care interventions intersect with cancer treatment efficacy. In this multicenter real-world cohort, systemic ATB exposure shortly before or during NACT was associated with lower odds of achieving favorable pathologic response, particularly the composite RCB-0/I endpoint. Although differences in pCR were more modest, ATB exposure was also associated with lower odds of RCB-0 after adjustment. The association with RCB-0/I persisted after adjustment for clinical, pathologic, and treatment-related factors, including global RDI and ECOG performance status, and was directionally consistent across the main IHC subtypes. These findings add to the limited clinical evidence evaluating ATB exposure as a potential host-related factor associated with neoadjuvant treatment response in BC.

A central issue in interpreting these results is confounding by indication. ATB exposure in this setting is not random and may reflect febrile neutropenia, documented infection, treatment-related complications, or greater clinical vulnerability. To address this concern, we incorporated ECOG performance status, the age-adjusted Charlson Comorbidity Index, comorbidity domains, and treatment-delivery variables. Baseline functional status and comorbidity burden were generally balanced according to ATB exposure; no prespecified non–ATB-related covariate modified the ATB OR by more than 10%, and ATB estimates remained stable in sensitivity analyses incorporating Charlson. These analyses reduce, but cannot eliminate, the possibility that the observed association was driven by baseline frailty or comorbidity.

Treatment delivery is particularly relevant because infection and its management may compromise chemotherapy intensity and scheduling. In our cohort, ATB-exposed patients had modestly higher rates of reduced global RDI, omitted cycles, and clinically relevant NACT prolongation. Reduced global RDI (<85%) was associated with lower odds of achieving RCB-0/I, consistent with prior evidence linking RDI to chemotherapy effectiveness [[38], [39], [40], [41]]. However, most patients exposed to ATBs maintained preserved treatment delivery, with more than 80% achieving global RDI ≥85%. Moreover, the association between ATB exposure and RCB-0/I persisted after adjustment for RDI, was essentially unchanged when RDI was modelled as a continuous variable and remained evident among patients with preserved global RDI. Therefore, measured treatment delivery does not appear to fully explain the ATB–response association, while residual confounding cannot be excluded.

The pattern of ATB exposure provides additional clinical context. Most exposed patients received a single ATB course and treatment was predominantly oral. Although febrile neutropenia was the most common individual indication, it accounted for only about one-third of ATB-exposed patients. Thus, ATB exposure was heterogeneous and, based on available descriptors, frequently occurred in the context of infectious complications managed within routine supportive care rather than being restricted to severe neutropenic events or intravenous treatment. Formal infection severity grading was not available in this retrospective real-world dataset; therefore, timing, route, duration, number of courses, and indication should be interpreted as objective clinical descriptors rather than as a validated severity scale.

Across IHC subtypes, ATB exposure was associated with numerically lower RCB-0/I rates. The largest absolute difference was observed in HER2-positive tumors, a subtype in which immune-dependent mechanisms may contribute to anti-HER2 efficacy. In luminal tumors, the significant reduction in RCB-0/I despite lower baseline chemosensitivity suggests that this association was not restricted to more immunogenic subtypes. In triple-negative disease, the magnitude of the difference was also clinically relevant, although it did not reach statistical significance. However, formal interaction testing was not significant, indicating that these subtype-specific findings should be interpreted as descriptive and hypothesis-generating. Overall, this pattern supports further exploration of host-related factors beyond intrinsic tumor characteristics.

The closest prior clinical evidence comes from a small neoadjuvant BC cohort (N = 120), with ATB exposure frequencies similar to those observed in our study; that cohort reported lower response rates and worse survival among ATB-exposed patients, with exploratory signals in HER2-positive disease [25]. Our larger multicenter cohort extends these observations using RCB-defined endpoints and detailed treatment-delivery assessment. Additional support comes from exploratory analyses of the I-SPY2 platform, in which ATB exposure was associated with higher residual cancer burden in BC [13], and from data linking antimicrobial exposure to decreased survival in triple-negative BC [42]. These studies support prospective evaluation of ATB exposure as a potential clinical cofactor.

These findings are biologically plausible in light of preclinical HER2-positive models showing that trastuzumab efficacy may partly depend on microbiota-induced immune stimulation. In these models, ATB-induced dysbiosis impaired CD4^+^ T-cell recruitment, granzyme B+ immune-cell activation, and dendritic-cell function, with lower α-diversity and reduced response [43]. This aligns with the immune-dependent activity of trastuzumab through CD4^+^ T cells, NK cells, and IL-12–producing dendritic cells [44].

More broadly, this pattern supports the hypothesis that ATBs may act not only as passive co-medications but as host-related clinical cofactors influencing antitumor efficacy. This expands the traditional focus on cytotoxic exposure and tumor biology [45], underscoring the need to integrate host-related factors such as microbiota integrity [[46], [47], [48]]. Classical pharmacokinetic interactions are unlikely to explain our results, as commonly used ATBs have no established clinically relevant interactions with taxanes, cyclophosphamide, or anthracyclines [[49], [50], [51]]. Thus, an indirect mechanism involving microbiota disruption and immune modulation remains plausible.

Mounting evidence suggests that the microbiota contributes to antitumor immunity [52]. Anthracyclines can induce immunogenic cell death, promoting dendritic-cell activation and T-cell priming [[53], [54], [55]]. Recent BC data further suggest that anthracycline-based NACT induces tumor-derived DNA fragments that engage innate immune sensors, with CA-rich DNA activating dendritic-cell cGAS–STING signaling and type I interferon responses linked to chemosensitivity [56]. Similarly, in the TONIC trial, short-course doxorubicin priming enhanced immune infiltration and upregulated immune-related genes, including interferon, TNF-α, and JAK–STAT pathways, improving subsequent PD-1 blockade efficacy in triple-negative BC [57].

These observations align with the immuno-oncology–microbiome axis, in which microbiota influence antigen presentation and immune activation [58]. Cyclophosphamide provides a relevant example: in preclinical models, epithelial damage allowed translocation of Gram-positive bacteria into lymphoid tissues, promoting pTH17 and TH1 responses involved in treatment efficacy; ATBs disrupted these pathways and reduced antitumor activity [59].

### 4.1 Limitations

This study has limitations. Its retrospective observational design precludes causal inference, and residual confounding remains possible despite multivariable adjustment and sensitivity analyses. ATB exposure may partly reflect infection severity, treatment-related complications, or unmeasured frailty rather than a direct effect of ATBs on tumor response. Although frailty-related baseline characteristics and treatment-delivery variables were incorporated, they cannot fully capture the clinical complexity of infection or deterioration during NACT. Formal infection-severity grading was unavailable; therefore, timing, route, duration, number of courses, and indication should be interpreted as clinical descriptors rather than as a validated severity scale. ATB exposure occurred predominantly during NACT, whereas pre-NACT-only exposure was uncommon, suggesting that ATB use mainly reflected intercurrent infections rather than systematic prophylaxis. Although exposure could be classified as pre-NACT, during NACT, or both, the small pre-NACT-only subgroup and unavailable exact dates for some outpatient prescriptions limited assessment of whether pre-treatment exposure, which could theoretically affect early microbiota-dependent immune priming, had a distinct association with response.

Other microbiota-modifying exposures, including corticosteroids, chemotherapy-induced diarrhea, proton-pump inhibitors, and concomitant medications, were not systematically captured. Microbiota composition and immune biomarkers were not assessed, limiting mechanistic inference. RDI analyses were restricted to standard anthracycline- and taxane-based regimens with adequate documentation; less common protocols were excluded because of low frequency or methodological limitations, which may affect generalizability. Most patients were treated before chemoimmunotherapy became standard in early triple-negative BC, so associations may differ with ICI-containing regimens. Finally, outcomes were limited to pathologic response, and EFS or OS were not assessed. Although pCR and low RCB are validated individual-level prognostic markers, trial-level surrogacy for long-term outcomes is imperfect [60]. Therefore, prospective studies incorporating predefined ATB exposure windows, infection-severity grading, microbiome and immune profiling, and survival endpoints are needed.

### 4.2 Conclusion

Antibiotic exposure within 30 days before or during NACT was associated with a lower likelihood of achieving optimal pathologic response, with a broadly consistent association across BC subtypes and a more modest but significant reduction in pCR after adjustment for clinical and treatment-related factors. Formal interaction testing did not support significant subtype-specific effect modification.

These exploratory findings should not discourage clinically indicated antibiotic use, which remains essential for managing infections during cytotoxic chemotherapy. Rather, they suggest that antibiotic exposure may represent a relevant clinical cofactor and a potential source of heterogeneity in studies using pathologic response as an endpoint. Prospective studies incorporating predefined antibiotic exposure windows, infection-severity assessment, microbiome and immune profiling, and survival endpoints are warranted to clarify whether this association reflects a causal effect, a marker of clinical complexity, or both.

## Data statement

The data collected and analyzed in this article are not publicly available due to ethical, legal, and institutional restrictions and because the dataset may contain indirectly identifiable patient information. De-identified individual participant data underlying the published results may be made available upon written and detailed request to the corresponding author (m.zalabardo@uma.es), subject to additional institutional and ethics approvals and execution of a data-use agreement.

## Ethical approval statement

The study was approved by the CEIm Provincial de Málaga (Comité de Ética de la Investigación Provincial de Málaga; Andalusian accredited Research Ethics Committee) (SICEIA-2024-001478). This retrospective observational study used routinely collected electronic health record data. The requirement for informed consent was waived by the Ethics Committee because the study involved no intervention, posed minimal risk to participants, and all data were de-identified in accordance with applicable data protection regulations.

## Previous presentations

Preliminary data from this study were presented at the 2024 San Antonio Breast Cancer Symposium; December 2024; San Antonio, Texas.

## Declaration of generative AI and AI-assisted technologies in the writing process

During the preparation of this work, the author(s) used ChatGPT (OpenAI) to improve language and readability. After using this tool/service, the author(s) reviewed and edited the content as needed and take full responsibility for the content of the publication. No generative AI was used to generate, modify, or interpret study data or results.

## Funding information

This work was supported by institutional resources for research coordination and statistical analysis provided by the Fundación Pública Andaluza para la Investigación de Málaga en Biomedicina y Salud (10.13039/100024201FIMABIS), Málaga, Spain (no grant number applicable). The funder had no role in the study design; data collection, analysis, or interpretation; writing of the report; or the decision to submit the article for publication.

## CRediT authorship contribution statement

**M. Zalabardo:** Conceptualization, Data curation, Formal analysis, Investigation, Methodology, Validation, Visualization, Writing – original draft, Writing – review & editing. **I. Fernández:** Data curation, Investigation, Writing – review & editing. **A. Girona:** Data curation, Investigation, Writing – review & editing. **Á. González:** Data curation, Investigation, Writing – review & editing. **J. Blanco:** Writing – original draft, Writing – review & editing. **Ó. Campos:** Data curation, Investigation, Writing – review & editing. **A. Sánchez-Muñoz:** Conceptualization, Methodology, Supervision, Writing – original draft, Writing – review & editing. **J.L. Onieva:** Formal analysis, Methodology, Software. **J.M. Jerez:** Formal analysis, Methodology, Software. **J. Pascual:** Data curation, Investigation, Writing – review & editing. **M.J. Bermejo:** Data curation, Investigation, Writing – review & editing. **B. Jiménez:** Data curation, Investigation, Writing – review & editing. **A. Márquez:** Data curation, Investigation, Writing – review & editing. **B.I. Pajares:** Data curation, Investigation, Writing – review & editing. **M. Domínguez:** Data curation, Investigation, Writing – review & editing. **A. Godoy-Ortiz:** Data curation, Investigation, Writing – review & editing. **T. Díaz:** Data curation, Investigation, Writing – review & editing. **I. Zarcos:** Data curation, Investigation, Writing – review & editing. **F. Carabantes:** Data curation, Investigation, Writing – review & editing. **E. Villar:** Data curation, Investigation, Writing – review & editing. **N. Ribelles:** Data curation, Investigation, Project administration, Resources, Validation, Writing – original draft, Writing – review & editing. **E. Alba:** Conceptualization, Project administration, Resources, Supervision, Validation, Writing – original draft, Writing – review & editing.

## Declaration of competing interest

The authors declare the following financial interests/personal relationships which may be considered as potential competing interests:M Zalabardo reports a relationship with Novartis that includes: funding grants, speaking and lecture fees, and travel reimbursement. M Zalabardo reports a relationship with Pfizer that includes: funding grants and travel reimbursement. M Zalabardo reports a relationship with Pierre Fabre SA that includes: speaking and lecture fees and travel reimbursement. M Zalabardo reports a relationship with AstraZeneca that includes: funding grants and speaking and lecture fees. M Zalabardo reports a relationship with Regeneron Pharmaceuticals Inc that includes: speaking and lecture fees. M Zalabardo reports a relationship with Nutricia Ltd that includes: consulting or advisory. M Zalabardo reports a relationship with Abbott Laboratories that includes: consulting or advisory. I Fernandez reports a relationship with Eli Lilly and Company that includes: funding grants. I Fernandez reports a relationship with Delical that includes: funding grants and travel reimbursement. A Girona reports a relationship with Nutricia Ltd that includes: funding grants and travel reimbursement. A Girona reports a relationship with Merck & Co Inc that includes: funding grants and travel reimbursement. A Girona reports a relationship with Roche that includes: funding grants and travel reimbursement. Á. González reports a relationship with AstraZeneca that includes speaking and lecture fees. Á. González reports a relationship with Pfizer that includes speaking and lecture fees. Á. González reports a relationship with Roche that includes speaking and lecture fees. Á. González reports a relationship with LEO Pharma that includes speaking and lecture fees. Á. González reports a relationship with Rovi that includes speaking and lecture fees. Á. Gonzalez reports a relationship with Novartis that includes: funding grants and travel reimbursement. Á. Gonzalez reports a relationship with Adamed that includes: funding grants and travel reimbursement. J Blanco reports a relationship with Roche that includes: funding grants and travel reimbursement. J Blanco reports a relationship with Celltrion, Inc. that includes: funding grants and travel reimbursement. J Pascual reports a relationship with AstraZeneca that includes: consulting or advisory, funding grants, non-financial support, speaking and lecture fees, and travel reimbursement. J Pascual reports a relationship with Novartis that includes: consulting or advisory, speaking and lecture fees, and travel reimbursement. J Pascual reports a relationship with Roche that includes: funding grants. J Pascual reports a relationship with Pfizer that includes: funding grants, non-financial support, speaking and lecture fees, and travel reimbursement. J Pascual reports a relationship with AstraZeneca that includes: consulting or advisory, funding grants, non-financial support, speaking and lecture fees, and travel reimbursement. J Pascual reports a relationship with Gilead Sciences Inc that includes: non-financial support, speaking and lecture fees, and travel reimbursement. J Pascual reports a relationship with Merck Sharp & Dohme Corp that includes: consulting or advisory. J Pascual reports a relationship with Pierre Fabre SA that includes: speaking and lecture fees. J Pascual reports a relationship with Eli Lilly and Company that includes: speaking and lecture fees. J Pascual reports a relationship with Stemline Therapeutics Inc that includes: consulting or advisory. A Godoy reports a relationship with Novartis that includes: consulting or advisory and speaking and lecture fees. A Godoy reports a relationship with AstraZeneca that includes: consulting or advisory and speaking and lecture fees. E Alba reports a relationship with Pfizer that includes: consulting or advisory and funding grants. E Alba reports a relationship with AstraZeneca that includes: consulting or advisory and funding grants. E Alba reports a relationship with Eli Lilly and Company that includes: consulting or advisory. E Alba reports a relationship with EXAS that includes: consulting or advisory and funding grants. E Alba reports a relationship with Bristol-Myers Squibb Company that includes: consulting or advisory. E Alba reports a relationship with Novartis that includes: funding grants. If there are other authors, they declare that they have no known competing financial interests or personal relationships that could have appeared to influence the work reported in this paper.
