# Supplementary material for: Association between antibiotic use and pathologic response to neoadjuvant chemotherapy in breast cancer: a multicentre retrospective cohort study
Source: Breast. 2026 Jun 11;88:104833. doi: 10.1016/j.breast.2026.104833 (PMC13280407; doi:10.1016/j.breast.2026.104833)
Supplement: Multimedia component 1 [file mmc1.pdf]

ESMO-GROW Checklist (completed) – Antibiotics and pathologic response to neoadjuvant chemotherapy in breast cancer

Manuscript: Association Between Antibiotic Use and Pathologic Response to Neoadjuvant Chemotherapy in Breast Cancer

Journal target: Annals of Oncology (Real-world data reporting).

Item	Recommendation (ESMO-GROW)	Reported?	Where addressed
1.1	Concisely include relevant key terms referring to the study type, study population, objectives, data sources and outcomes, depending on the study. Consider including the terms 'real-world' or 'observational'	Yes, fully	Title; Abstract (Background/Patients and methods)
2.1	Explain the scientific rationale for the research question(s), providing concise background information on previous core evidence from systematic reviews, meta-analyses, clinical trials and/or real-world evidence studies	Yes, fully	Introduction (paragraphs 1–4)
2.2	Identify the gaps in evidence and explain why and how they can be suitably addressed by real-world evidence research. Specify the new evidence that is expected from the current study	Yes, fully	Introduction (paragraphs 4–5)
2.3	Briefly introduce the aim(s) of the study	Yes, fully	Introduction (final sentence)
3.1	Provide the study research question(s) including a description of the patients or the object under study and the target outcome(s)	Yes, fully	Methods: Study Design; Outcomes

3.2	Provide the study objective(s) and consider classifying the type of research as descriptive and/or analytical (explanatory or predictive)	Yes, fully	Methods: Study Design; Statistical Analysis
3.3	Provide relevant information to describe and classify the study design used to address the research question	Yes, fully	Methods: Study Design
3.4	Give a clear definition of the eligibility criteria used to select the patients or objects under study, particularly regarding cancer-related aspects	Yes, fully	Methods: Population and Data Source (inclusion/exclusion)
3.5	Report the specific type and purpose of real-world data source(s) used, providing a detailed description and the reason(s) why the source was considered appropriate for the study objectives	Yes, fully	Methods: Population and Data Source (Galen EHR and modules)
3.6	When multiple real-world data sources are used, provide details on interoperability, including identification of duplicated cases or data linkage from separate databases	Not applicable	Single integrated EHR source (Galen)
3.7	Provide details and timings of source and study data management. Consider specifying methods of raw data collection, updates and completeness, data extraction, cleaning and/or quality controls and validation	Yes, fully	Methods: Population and Data Source (data extraction); Supplementary Methods
3.8	Provide core details on database and/or study	Yes, fully	Methods: Ethics; Supplementary

	registration, governance, ownership, metadata and full data accessibility in the main text or supplementary material		Methods: Data availability statement (if included) / on request
3.9	Identify the data source of each core variable, its definition, if the variable was derived or coded, and describe how the derivation or coding was conducted and validated.	Yes, partially	Methods: Antibiotic Exposure; Variables and Measures; RDI (definitions)
3.10	Specify the time points of core variables in relation to the cancer disease trajectory	Yes, fully	Methods: Antibiotic Exposure (30-day pre-NACT through surgery); Baseline at diagnosis
3.11	Provide a complete list of core variables included in the study. Variables can be grouped as baseline characteristics, exposure and outcomes or endpoints	Yes, fully	Methods: Variables and Measures (covariates list)
3.12	For biomarker-related studies, provide details on biomarker description, timing, and methods of assessment and analytical validation Statistical analysis and artificial intelligence methods	Not applicable	No biomarker assay study
3.13	Summarise the main aspects of the statistical analysis	Yes, fully	Methods: Statistical Analysis
3.14	When applicable, provide details on the pre-planned sample size requirements and power of the study	Not applicable	Retrospective cohort (no a priori sample size/power)
3.15	Specify the pre-planned strategies to identify and mitigate the main sources of bias	Yes, fully	Methods: Confounding/collinearity strategy; Discussion: Limitations

3.16	Clearly distinguish prespecified from post hoc analyses, especially for subgroup analyses	Yes, fully	Methods: prespecified endpoints; Results: subtype analyses (clarify prespecified vs post hoc)
3.17	Provide information on internal and external validity, as well as any sensitivity analyses	Yes, fully	Methods/Results: sensitivity analyses (RDI continuous vs categorical)
3.18	For analytical studies, the full version of the statistical analysis plan should be provided in the supplementary material, including a brief explanation of any amendments	Yes, fully	Methods: Statistical Analysis (main specs); Supplementary Methods (expanded details)
3.19	When applicable, specify which machine learning, deep learning or alternative artificial intelligence method has been used	Not applicable	No AI/ML methods
3.20	When reporting real-world data analysis with artificial intelligence (e.g. machine learning and deep learning) algorithms, include comprehensive aspects on data pre-processing techniques, feature engineering strategies and model development	Not applicable	No AI/ML methods
3.21	Address the artificial intelligence model explainability and interpretability, and present the plan for integration into clinical practice, if applicable	Not applicable	No AI/ML methods
3.22	When applicable, briefly describe the multidisciplinary team required for the study and explain how these needs were met	Not applicable	No AI/ML methods (multidisciplinary AI team not relevant)

4.1	Provide number of cases excluded or nonparticipating and reasons at each stage of sample selection, as well as numbers lost to follow-up. Compare the cases excluded with those included in the analyses. Illustrate this with a flowchart	Yes, fully	Results: cohort selection; GROW flowchart (this file); Supplementary Figure S1
4.2	Describe the baseline characteristics of the cases included (e.g. clinico-demographic and tumour characteristics). The baseline characteristics of different groups under analysis should be compared, if applicable	Yes, fully	Results: Baseline characteristics (Table 1)
4.3	Report the results of the primary analysis of study outcomes. Briefly describe the results of exploratory analyses if relevant (prespecified and/or post hoc). Provide details of how readers can access the full results	Yes, fully	Results: primary/secondary outcomes; multivariable models; figures/tables
5.1	Summarise the core results that address the primary research question(s) and objectively discuss the data in relation to the best available evidence on the topic. Avoid a convenient selection of literature to support a point	Yes, fully	Discussion: paragraphs 1–6
5.2	Discuss the strengths and limitations of the current study, including the main biases, how the strategies applied contributed to bias avoidance or mitigation, and, if applicable, in	Yes, fully	Discussion: strengths/Limitations section

	which direction the authors estimate that residual bias may influence the core results of the study		
5.3	Discuss the generalisability of the study results and their potential implications for clinical practice, health policies or public health and for the generation of hypotheses for future research Conclusions	Yes, fully	Discussion: generalisability/clinical implications
5.4	Provide a balanced summary of core results relating to the primary research question and the main implications for clinical practice, health policies and/or public health. Suggest further research considering the remaining unmet needs and limitations from the reported study	Yes, fully	Conclusion
6.1	Specify all relevant study sponsorship(s) as well as direct and/or indirect or in-kind funding	Yes, fully	Funding statement (end matter)
6.2	Specify all relevant acknowledgements, author disclosures, individual contributions and other final considerations as per journal regulations	Yes, fully	Acknowledgements; Disclosures/Author contributions (submission system or end matter)
