# Supplementary material for: Association between antibiotic use and pathologic response to neoadjuvant chemotherapy in breast cancer: a multicentre retrospective cohort study
Source: Breast. 2026 Jun 11;88:104833. doi: 10.1016/j.breast.2026.104833 (PMC13280407; doi:10.1016/j.breast.2026.104833)
Supplement: Multimedia component 2 [file mmc2.docx]

**Supplementary Material**
**Association Between Antibiotic Use and Pathologic Response to Neoadjuvant Chemotherapy in Breast Cancer**

**Contents**

1. **Supplementary Methods**
   1.1. Data source, governance, registration, and accessibility

1.2. Baseline comorbidity assessment
1.3. Rationale for exclusion of certain regimens from RDI analysis

1.4. Supplementary Statistical Analysis Plan (SAP)

1. **Supplementary Tables**

Table S1. Baseline comorbidity profile by antibiotic exposure
Table S2. Antibiotic combinations among exposed patients
Table S3. Complete distribution of chemotherapy regimens by antibiotic exposure
Table S4. Original chemotherapy regimens categorized by final treatment groups

Table S5. Covariate-by-covariate assessment of potential confounding of the ATB–response association
Table S6. Summary of sensitivity analyses for the association between ATB exposure and pathologic response

1. **Supplementary Figures**
   Figure S1. Patient selection flowchart (GROW)
   Figure S2. Distribution of clinical indications for antibiotic use
   Figure S3. Distribution of optimal pathologic response (RCB-0/I) by IHC subtype and antibiotic exposure
   Figure S4. Distribution of complete pathologic response (RCB-0) by IHC subtype and antibiotic exposure
2. **Supplementary Methods**
   1. **Data source, governance, registration, and accessibility**

**Data source and governance:** Data were obtained from the Galen electronic health record used at the participating hospitals. The dataset contains retrospective clinical information that may be indirectly identifiable; data were anonymized for analysis, and access was granted under institutional governance and data-protection policies to authorized study personnel only.

**Registration:** The study was not prospectively registered (retrospective observational design).

**Data accessibility:** Public data sharing was not covered in the original protocol; therefore, the de-identified dataset is not publicly available, and access may be considered upon reasonable request subject to additional institutional and ethical approvals and data-sharing agreements.

- 1. **Baseline comorbidity assessment**

Baseline comorbidity was abstracted from clinical records at the start of neoadjuvant chemotherapy. Comorbidity burden was summarized using the Charlson Comorbidity Index, including age. In addition, prespecified clinical domains were recorded to characterize baseline frailty and potential confounding by indication.

The following domains were considered:

- **Diabetes/metabolic disease:** including diabetes mellitus, morbid obesity when explicitly documented, or other clinically relevant metabolic disease
- **Cardiovascular disease:** including ischemic heart disease, prior myocardial infarction, heart failure, clinically relevant arrhythmia, valvular heart disease, or peripheral vascular disease.
- **Pulmonary disease**: including chronic obstructive pulmonary disease, moderate-to-severe asthma, interstitial lung disease, bronchiectasis, pulmonary hypertension, or chronic oxygen therapy.
- **Renal disease**: including chronic kidney disease, chronic renal failure, dialysis, or kidney transplantation.
- **Hepatic disease:** including cirrhosis, chronic hepatitis B or C, nonalcoholic steatohepatitis with fibrosis, or portal hypertension.
- **Neurological disease**: including stroke or transient ischemic attack, dementia, Parkinson disease, multiple sclerosis, amyotrophic lateral sclerosis, epilepsy, or other clinically relevant chronic neurological disorders.
- **Autoimmune/inflammatory disease:** including systemic autoimmune diseases such as lupus, Sjögren syndrome, systemic sclerosis, or vasculitis; inflammatory arthritis, including rheumatoid arthritis, spondyloarthritis, psoriatic arthritis, polymyalgia rheumatica, or giant cell arteritis; inflammatory bowel disease, including Crohn disease or ulcerative colitis; moderate-to-severe psoriasis, particularly with joint involvement or systemic treatment; clinically relevant sarcoidosis; myasthenia gravis; or other clinically relevant chronic immune-mediated inflammatory disorders**.**
- **Psychiatric disorder**: including depression under active treatment, bipolar disorder, schizophrenia, psychotic disorder, severe psychiatric disease, or chronic psychiatric treatment.

Isolated hypertension, stable hypothyroidism under replacement therapy, and isolated dyslipidemia were not considered major comorbidity domains.

**1.3 Rationale for the Exclusion of Certain Regimens From RDI Analysis**

The detailed analysis of relative dose intensity (RDI) was restricted to patients treated with conventional neoadjuvant regimens based on anthracyclines, taxanes, and cyclophosphamide, with or without anti-HER2 agents.

Several chemotherapeutic components were excluded from this analysis, as they were administered to a small proportion of the cohort (40 patients [3.0%]) and presented methodological limitations:

- Agents such as carboplatin, cisplatin, or fluoropyrimidines (eg, 5-fluorouracil) were used in regimens uncommon in our current neoadjuvant practice without immunotherapy.
- In combination regimens such as carboplatin-paclitaxel, only the paclitaxel component (taxane) was considered in the overall RDI analysis. Carboplatin was excluded because its dosing relies on pharmacokinetic parameters (eg, creatinine clearance), which were not consistently available.

These exclusions were made to preserve methodological homogeneity, minimize bias from missing data, and ensure the robustness of RDI calculations.

In addition, fixed-dose anti-HER2 therapies (such as trastuzumab and pertuzumab) were excluded from RDI analysis, as their dosing is standardized, rarely adjusted in clinical practice, and no validated methods currently exist for calculating RDI for monoclonal antibodies.

**1.4 Supplementary Statistical Analysis Plan (SAP)**

**Objectives and endpoints**

To evaluate whether systemic antibiotic (ATB) exposure is associated with pathologic response after neoadjuvant chemotherapy (NACT) in early-stage breast cancer. Primary endpoint: RCB-0/I vs RCB-II/III. Secondary endpoint: RCB-0 vs RCB-I/II/III.

**Exposure**

ATB exposure was defined as receipt of ≥1 systemic antibiotic course within 30 days before NACT initiation or during NACT until surgery (yes vs no).

**Cohort identification and data handling**

Patients were identified from the shared electronic health record system (Galen). Patients without surgery, treated with immune checkpoint inhibitor–based regimens, or with insufficient documentation of chemotherapy dosing or ATB exposure were excluded to minimize misclassification. Antibiotic exposure and chemotherapy dosing were verified across available electronic sources.

**Candidate covariates**

Candidate covariates were prespecified on clinical grounds:

- **Clinical:** age group, menopausal status, ECOG performance status, clinical stage (TNM), and age-adjusted Charlson Comorbidity Index.
- **Pathologic:** histologic grade, Ki-67 category, histology, and immunohistochemical (IHC) subtype.
- **Treatment-related:** NACT regimen, anthracycline use, and global relative dose intensity (RDI).
- **ATB-related**: number of courses, route of administration, and clinical indication.

**Descriptive analyses**

Categorical variables were summarized as No. (%) and compared using χ² or Fisher exact tests, as appropriate. Continuous variables were summarized as median (IQR) or mean (SD), as appropriate, and compared using Wilcoxon rank-sum tests. Two-sided P<0.05 was considered statistically significant.

**Missing data**

Missing covariates were imputed to the modal category.

**Primary multivariable models**

Multivariable logistic regression models estimated adjusted odds ratios (ORs) and 95% confidence intervals (CIs) for both endpoints. Global RDI was included a priori. ECOG performance status was forced into the final models to account for baseline functional status. Model selection used bidirectional stepwise selection based on the Akaike Information Criterion.

**Collinearity and confounding**

Collinearity was assessed using the variance inflation factor (VIF); VIF≥5 indicated problematic multicollinearity. Potential confounding was explored by comparing the crude ATB OR with models adjusted individually for each core covariate; a >10% change in the ATB OR indicated relevant confounding. ATB burden descriptors (number of courses, route, indication) were not entered as separate covariates due to collinearity with the main exposure.

**Prespecified subgroup and sensitivity analyses**

Models were prespecified to be repeated within grouped IHC subtypes (luminal, HER2-positive, and triple-negative). Sensitivity analyses compared RDI modeled as <85% vs ≥85% and as a continuous percentage, models restricted to patients with preserved global RDI (≥85%), and models incorporating the age-adjusted Charlson Comorbidity Index to assess robustness to treatment delivery and baseline comorbidity/frailty.

**Interaction testing**

Formal interaction testing was performed by adding an antibiotic exposure × grouped IHC subtype interaction term to the overall model and comparing nested models using a likelihood-ratio χ² test.

**Model evaluation**

Calibration was assessed using the Hosmer–Lemeshow test. The incremental contribution of ATB exposure was evaluated using a likelihood ratio test comparing the full model with a reduced model excluding ATB.

**Software**

All analyses were performed in R (version 4.3.1). Data were analyzed between January 2024 and July 2024.

**Amendments**

Amendments: After initial data review, chemotherapy regimen was excluded from overall models due to collinearity with IHC subtype (VIF>20) and assessed in prespecified subtype-stratified models.

1. **Supplementary Tables**

**Table S1. Baseline comorbidity profile by antibiotic exposure**

|  | **OVERALL**  **(*N*=1316), *N* (%)** | **NO ANTIBIOTIC (*N*=800), *N* (%)** | **ANTIBIOTIC (*N*=516), *N* (%)** | ***P*** |
| --- | --- | --- | --- | --- |
| **Age-adjusted Charlson Comorbidity Index** | | | | |
| 0 | 613 (46.6) | 375 (46.9) | 238 (46.1) | 0.95 |
| 1 | 331 (25.1) | 199 (24.9) | 132 (25.6) |  |
| ≥2 | 372 (28.3) | 226 (28.2) | 146 (28.3) |  |
| **Comorbidity domains** | | | | |
| Diabetes/metabolic disease | 99 (7.5) | 59 (7.4) | 40 (7.8) | 0.83 |
| Cardiovascular disease | 57 (4.3) | 37 (4.6) | 20 (3.9) | 0.58 |
| Pulmonary disease | 65 (4.9) | 33 (4.1) | 32 (6.2) | 0.09 |
| Renal disease | 15 (1.1) | 12 (1.5) | 3 (0.6) | 0.18 |
| Hepatic disease | 11 (0.8) | 10 (1.3) | 1 (0.2) | 0.06 |
| Neurological disease | 42 (3.2) | 24 (3.0) | 18 (3.5) | 0.63 |
| Autoimmune/inflammatory disease | 84 (6.4) | 55 (6.9) | 29 (5.6) | 0.42 |
| Psychiatric disorder | 77 (5.9) | 64 (8.0) | 13 (2.5) | <0.01 |
| **Number of comorbidity domains** | | | | |
| 0 | 1010 (76.7) | 610 (76.3) | 400 (77.5) | 0.06 |
| 1 | 234 (17.8) | 137 (17.1) | 97 (18.8) |  |
| ≥2 | 72 (5.5) | 53 (6.6) | 19 (3.7) |  |

Data are presented as No. (%). P values were calculated using χ² or Fisher exact tests, as appropriate. Individual comorbidity domains are reported as the number of patients with at least one condition in each domain; a given patient could contribute to more than one domain. The grouped comorbidity burden variable summarizes the number of affected domains per patient (0, 1, or ≥2). Definitions of comorbidity domains are provided in the Methods.

**Table S2. Antibiotic Combinations Among Exposed Patients.**

| **ANTIBIOTIC (*N*=516)** | ***N* (%)** |
| --- | --- |
| Aminoglucosides + Nitroimidazols | 1 (0.2%) |
| Beta-lactams | 234 (45.3%) |
| Beta-lactams + Lincosamides | 3 (0.6%) |
| Beta-lactams + Macrolides | 10 (1.9%) |
| Beta-lactams + Nitroimidazols + Aminoglucosides | 1 (0.2%) |
| Beta-lactams + Oxazolidinones | 1 (0.2%) |
| Beta-lactams + Phosphonates | 13 (2.5%) |
| Beta-lactams + Quinolones | 114 (22.1%) |
| Beta-lactams + Quinolones + Aminoglucosides | 1 (0.2%) |
| Beta-lactams + Quinolones + Aminoglucosides + Nitroimidazols | 2 (0.4%) |
| Beta-lactams + Quinolones + Lincosamides | 1 (0.2%) |
| Beta-lactams + Quinolones + Phosphonates | 7 (1.4%) |
| Beta-lactams + Sulfonamides | 1 (0.2%) |
| Lincosamides | 4 (0.8%) |
| Lincosamides + Quinolones | 1 (0.2%) |
| Macrolides | 24 (4.7%) |
| Macrolides + Phosphonates | 1 (0.2%) |
| Nitroimidazols | 1 (0.2%) |
| Oxazolidinones | 1 (0.2%) |
| Phosphonates | 30 (5.8%) |
| Phosphonates + Lincosamides | 1 (0.2%) |
| Phosphonates + Macrolides | 1 (0.2%) |
| Quinolones | 52 (10.1%) |
| Quinolones + Lincosamides | 3 (0.6%) |
| Quinolones + Macrolides | 2 (0.4%) |
| Quinolones + Nitroimidazols | 1 (0.2%) |
| Quinolones + Phosphonates | 1 (0.2%) |
| Quinolones + Tetracyclines | 1 (0.2%) |
| Tetracyclines | 3 (0.6%) |

Percentages are based on the 516 patients who received at least one antibiotic course within 30 days before or during neoadjuvant chemotherapy. “Other antibiotics” include antibiotic regimens used in ≤1% of patients, such as β-lactams with oxazolidinones, sulfonamides, or multiple agents.

Values are presented as No. (%).

**Table S3. Complete Distribution of Chemotherapy Regimens by Antibiotic Exposure**

| **Chemotherapy Regimen** | **TOTAL**  **(*N*=1316), *N* (%)** | **NO ANTIBIOTIC (*N*=800), *N* (%)** | **YES ANTIBIOTIC**  **(*N*=516), *N* (%)** |
| --- | --- | --- | --- |
| AC | 7 (0.5%) | 4 (0.5%) | 3 (0.6%) |
| AC (DD)-DOCETAXEL | 3 (0.2%) | 1 (0.1%) | 2 (0.4%) |
| AC (DD)-DOCETAXEL-TRASTUZUMAB-PERTUZUMAB | 2 (0.1%) | 1 (0.1%) | 1 (0.2%) |
| AC-DOCETAXEL | 298 (22.6%) | 189 (23.2%) | 109 (21.1%) |
| AC-DOCETAXEL-LAPATINIB | 3 (0.2%) | 3 (0.4%) | 0 (0.0%) |
| AC-DOCETAXEL-PACLITAXEL | 1 (0.1%) | 1 (0.1%) | 0 (0.0%) |
| AC-DOCETAXEL-TRASTUZUMAB | 80 (6.1%) | 55 (6.9%) | 25 (4.8%) |
| AC-DOCETAXEL-TRASTUZUMAB-PERTUZUMAB | 98 (7.4%) | 60 (7.4%) | 38 (7.4%) |
| AC-DOCETAXEL/PACLITAXEL | 13 (1.0%) | 4 (0.5%) | 9 (1.7%) |
| AC-DOCETAXEL/PACLITAXEL-TRASTUZUMAB | 3 (0.2%) | 2 (0.2%) | 1 (0.2%) |
| AC-DOCETAXEL/TC | 1 (0.1%) | 1 (0.1%) | 0 (0.0%) |
| AC-NABPACLITAXEL | 1 (0.1%) | 1 (0.1%) | 0 (0.0%) |
| AC-NABPACLITAXEL-TRASTUZUMAB | 1 (0.1%) | 0 (0.0%) | 1 (0.2%) |
| AC-PACLITAXEL | 428 (32.4%) | 253 (31.6%) | 175 (33.2%) |
| AC-PACLITAXEL-TRASTUZUMAB | 107 (8.1%) | 56 (7.0%) | 51 (9.9%) |
| AC-PACLITAXEL-TRASTUZUMAB-PERTUZUMAB | 164 (12.4%) | 104 (12.9%) | 60 (11.6%) |
| AC-TC | 5 (0.4%) | 5 (0.6%) | 0 (0.0%) |
| CARBOPLATIN-DOCETAXEL-AC | 15 (1.1%) | 10 (1.2%) | 5 (1.0%) |
| CARBOPLATIN-DOCETAXEL-TRASTUZUMAB | 2 (0.2%) | 1 (0.1%) | 1 (0.2%) |
| CARBOPLATIN-DOCETAXEL-TRASTUZUMAB-PERTUZUMAB | 10 (0.8%) | 6 (0.7%) | 4 (0.8%) |
| CARBOPLATIN-PACLITAXEL-AC | 4 (0.3%) | 4 (0.5%) | 0 (0.0%) |
| CARBOPLATIN-PACLITAXEL-TRASTUZUMAB-PERTUZUMAB | 8 (0.6%) | 4 (0.5%) | 4 (0.8%) |
| DOCETAXEL | 1 (0.1%) | 1 (0.1%) | 0 (0.0%) |
| DOCETAXEL-TRASTUZUMAB-PERTUZUMAB | 4 (0.3%) | 3 (0.4%) | 1 (0.2%) |
| FEC-DOCETAXEL | 1 (0.1%) | 1 (0.1%) | 0 (0.0%) |
| PACLITAXEL | 5 (0.4%) | 3 (0.4%) | 2 (0.4%) |
| PACLITAXEL-TRASTUZUMAB | 1 (0.1%) | 0 (0.0%) | 1 (0.2%) |
| PACLITAXEL-TRASTUZUMAB-PERTUZUMAB | 9 (0.7%) | 6 (0.7%) | 3 (0.6%) |
| TC | 27 (2.0%) | 16 (2.0%) | 11 (2.1%) |
| TC-TRASTUZUMAB | 2 (0.2%) | 0 (0.0%) | 2 (0.4%) |
| TC-TRASTUZUMAB-PERTUZUMAB | 5 (0.4%) | 1 (0.1%) | 4 (0.8%) |
| TRASTUZUMAB-PERTUZUMAB | 7 (0.5%) | 4 (0.5%) | 3 (0.6%) |

This table lists all individual neoadjuvant chemotherapy regimens administered in the full cohort, stratified by antibiotic exposure. Immunotherapy-containing regimens were excluded from analysis.

Abbreviations: AC, doxorubicin (or epirubicin) plus cyclophosphamide; DD, dose-dense; TC, docetaxel plus cyclophosphamide; CT + dHER2, chemotherapy plus double anti-HER2 blockade (trastuzumab and pertuzumab); CT + sHER2, chemotherapy plus single-agent anti-HER2 therapy (trastuzumab alone).

**Table S4. Original Chemotherapy Regimens Categorized by Final Treatment Groups Used in Statistical Analysis**

| **Chemotherapy Group** | **Original Chemotherapy Scheme** | ***N* (%)** |
| --- | --- | --- |
| AC-DTX (*N* = 321) | AC-DOCETAXEL | 298 (92.8%) |
|  | AC-DOCETAXEL/PACLITAXEL | 13 (4.0%) |
|  | AC-TC | 5 (1.6%) |
|  | AC (DD)-DOCETAXEL | 3 (0.9%) |
|  | AC-DOCETAXEL-PACLITAXEL | 1 (0.3%) |
|  | AC-DOCETAXEL/TC | 1 (0.3%) |
| AC-PTX (*N* = 429) | AC-PACLITAXEL | 428 (99.8%) |
|  | AC-NABPACLITAXEL | 1 (0.2%) |
| CT + dual HER2 blockade (*N* = 307) | AC-PACLITAXEL-TRASTUZUMAB-PERTUZUMAB | 164 (53.4%) |
|  | AC-DOCETAXEL-TRASTUZUMAB-PERTUZUMAB | 98 (31.9%) |
|  | CARBOPLATIN-DOCETAXEL-TRASTUZUMAB-PERTUZUMAB | 10 (3.3%) |
|  | PACLITAXEL-TRASTUZUMAB-PERTUZUMAB | 9 (2.9%) |
|  | CARBOPLATIN-PACLITAXEL-TRASTUZUMAB-PERTUZUMAB | 8 (2.6%) |
|  | TRASTUZUMAB-PERTUZUMAB | 7 (2.3%) |
|  | TC-TRASTUZUMAB-PERTUZUMAB | 5 (1.6%) |
|  | DOCETAXEL-TRASTUZUMAB-PERTUZUMAB | 4 (1.3%) |
|  | AC (DD)-DOCETAXEL-TRASTUZUMAB-PERTUZUMAB | 2 (0.7%) |
| CT + single HER2 blockade (*N* = 199) | AC-PACLITAXEL-TRASTUZUMAB | 107 (53.8%) |
|  | AC-DOCETAXEL-TRASTUZUMAB | 80 (40.2%) |
|  | AC-DOCETAXEL-LAPATINIB | 3 (1.5%) |
|  | AC-DOCETAXEL/PACLITAXEL-TRASTUZUMAB | 3 (1.5%) |
|  | CARBOPLATIN-DOCETAXEL-TRASTUZUMAB | 2 (1.0%) |
|  | TC-TRASTUZUMAB | 2 (1.0%) |
|  | AC-NABPACLITAXEL-TRASTUZUMAB | 1 (0.5%) |
|  | PACLITAXEL-TRASTUZUMAB | 1 (0.5%) |
| Other chemotherapy regimens (*N* = 60) | TC | 27 (45.0%) |
|  | CARBOPLATIN-DOCETAXEL-AC | 15 (25.0%) |
|  | AC | 7 (11.7%) |
|  | PACLITAXEL | 5 (8.3%) |
|  | CARBOPLATIN-PACLITAXEL-AC | 4 (6.7%) |
|  | DOCETAXEL | 1 (1.7%) |
|  | FEC-DOCETAXEL | 1 (1.7%) |

Original chemotherapy regimens are categorized by backbone agents and HER2 blockade strategy. Column percentages are calculated within each group.

Abbreviations**:** AC, doxorubicin and cyclophosphamide; DTX, docetaxel; TC, docetaxel and cyclophosphamide; DD, dose-dense; PTX, paclitaxel; CT, chemotherapy; FEC, 5-fluorouracil, epirubicin, and cyclophosphamide.

**Table S5. Covariate-by-covariate assessment of potential confounding of the ATB–response association**

| **Covariate individually added** | **ATB OR for RCB-0/I** | **% change from ATB OR** | **ATB OR for RCB-0** | **% change from ATB OR** |
| --- | --- | --- | --- | --- |
| Age group | 0.61 | 0.01% | 0.80 | 0.36% |
| Menopausal status | 0.61 | 0.12% | 0.80 | 0.30% |
| ECOG performance status | 0.60 | 0.07% | 0.80 | 0.08% |
| Age-adjusted Charlson Comorbidity Index | 0.60 | 0.17% | 0.80 | 0.11% |
| Stage (TNM) | 0.60 | 0.14% | 0.80 | 0.28% |
| Tumor subtype | 0.55 | 8.92% | 0.77 | 3.51% |
| Histological grade | 0.59 | 2.30% | 0.78 | 2.86% |
| Ki-67 index | 0.60 | 0.89% | 0.80 | 0.33% |
| Histological type | 0.60 | 1.42% | 0.80 | 0.65% |
| Global relative dose intensity | 0.62 | 1.78% | 0.80 | 0.40% |
| Treatment with anthracyclines | 0.61 | 0.29% | 0.80 | 0.05% |

Data show the ATB OR after adding each prespecified non–ATB-related covariate individually to the crude ATB–response model. Percentage change was calculated relative to the crude ATB OR; >10% was prespecified as relevant confounding. ATB-related descriptors were excluded because they were considered components of the exposure construct.

Abbreviations: ATB, antibiotic exposure; ECOG, Eastern Cooperative Oncology Group; IHC, immunohistochemical; OR, odds ratio; RCB, residual cancer burden; RDI, relative dose intensity; TNM, tumor–node–metastasis.

**Table S6. Summary of sensitivity analyses for the association between ATB exposure and pathologic response**

| **Sensitivity analysis** | **Model feature** | **ATB OR (95% CI), RCB-0/I** | ***P*** | **ATB OR (95% CI), RCB-0** | ***P*** |
| --- | --- | --- | --- | --- | --- |
| Global RDI as continuous covariate | Global RDI modeled as continuous percentage instead of <85% vs ≥85% | 0.55 (0.43–0.71) | <0.001 | 0.74 (0.57–0.97) | 0.03 |
| Charlson sensitivity model | Age group replaced by age-adjusted Charlson Comorbidity Index; ECOG retained | 0.56 (0.43–0.72) | <0.001 | 0.74 (0.57–0.97) | 0.03 |
| Restricted to global RDI ≥85% | Analysis restricted to patients with preserved global RDI | 0.58 (0.45–0.76) | <0.001 | 0.79 (0.59–1.05) | 0.11 |

This table summarizes sensitivity analyses performed to assess the robustness of the ATB–response association to alternative RDI modeling, baseline comorbidity/frailty adjustment, and preserved treatment delivery. The final multivariable model with ECOG forced is shown in Figure 1. In the Charlson sensitivity model, age group was replaced by the age-adjusted Charlson Comorbidity Index because of collinearity. ORs <1 indicate lower odds of achieving the corresponding pathologic response among ATB-exposed patients.

Abbreviations: ATB, antibiotic exposure; CI, confidence interval; ECOG, Eastern Cooperative Oncology Group; OR, odds ratio; RCB, residual cancer burden; RDI, relative dose intensity.

**3. Supplementary Figures**

**Figure S1. Patient Selection Flowchart (GROW)**

| **GROW Flowchart – Case selection and analysis groups**  Data sources: Galen shared EHR (3 hospitals) + inpatient prescribing/administration records +  oncology day-unit charts + outpatient prescribing module |
| --- |
| ↓ |
| **Identified in Galen database**  Women with localized breast cancer initiating NACT (Jan 2009–Jan 2024)  *N* = 1870 |
| ↓ |
| **Excluded (*N* = 554)**   - Lost to follow-up before surgery: *N* = 194 - Immunotherapy/ICI-based regimens: *N* = 28 - Incomplete/undocumented dosing or antibiotic exposure: *N* = 332 |
| ↓ |
| **Final analytic cohort**  *N* = 1316 |
| ↓ |
| \| **Antibiotic exposure**  ≥1 systemic course within 30 days pre-NACT or during NACT until surgery  *N* = 516  RCB-0/I: 188 (36.4%)  RCB-0: 143 (27.7%) \| **Unexposed**  No systemic antibiotics in exposure window  *N* = 800  RCB-0/I: 389 (48.6%)  RCB-0: 259 (32.4%) \| \| --- \| --- \| |
| ↙ ↘ |
| **Primary analysis: multivariable logistic regression**  Endpoints: RCB-0/I vs RCB-II/III (primary); RCB-0 vs RCB-I/II/III (secondary)  Adjusted for clinical, pathologic, and treatment factors (including global RDI) |

Patients with early breast cancer (BC) who received neoadjuvant chemotherapy (NACT) between January 2009 and January 2024 were assessed for eligibility. Of 1870 patients identified, 554 were excluded due to loss to follow-up before surgery (*N*=194), receipt of immune checkpoint inhibitor (ICI)-based regimens (*N*=28), or incomplete data (*N*=332), resulting in a final cohort of 1316 patients included in the analysis.

BC, breast cancer; NACT, neoadjuvant chemotherapy; ICI, immune checkpoint inhibitor.

**Figure S2. Distribution of Clinical Indications for Antibiotic Use.**


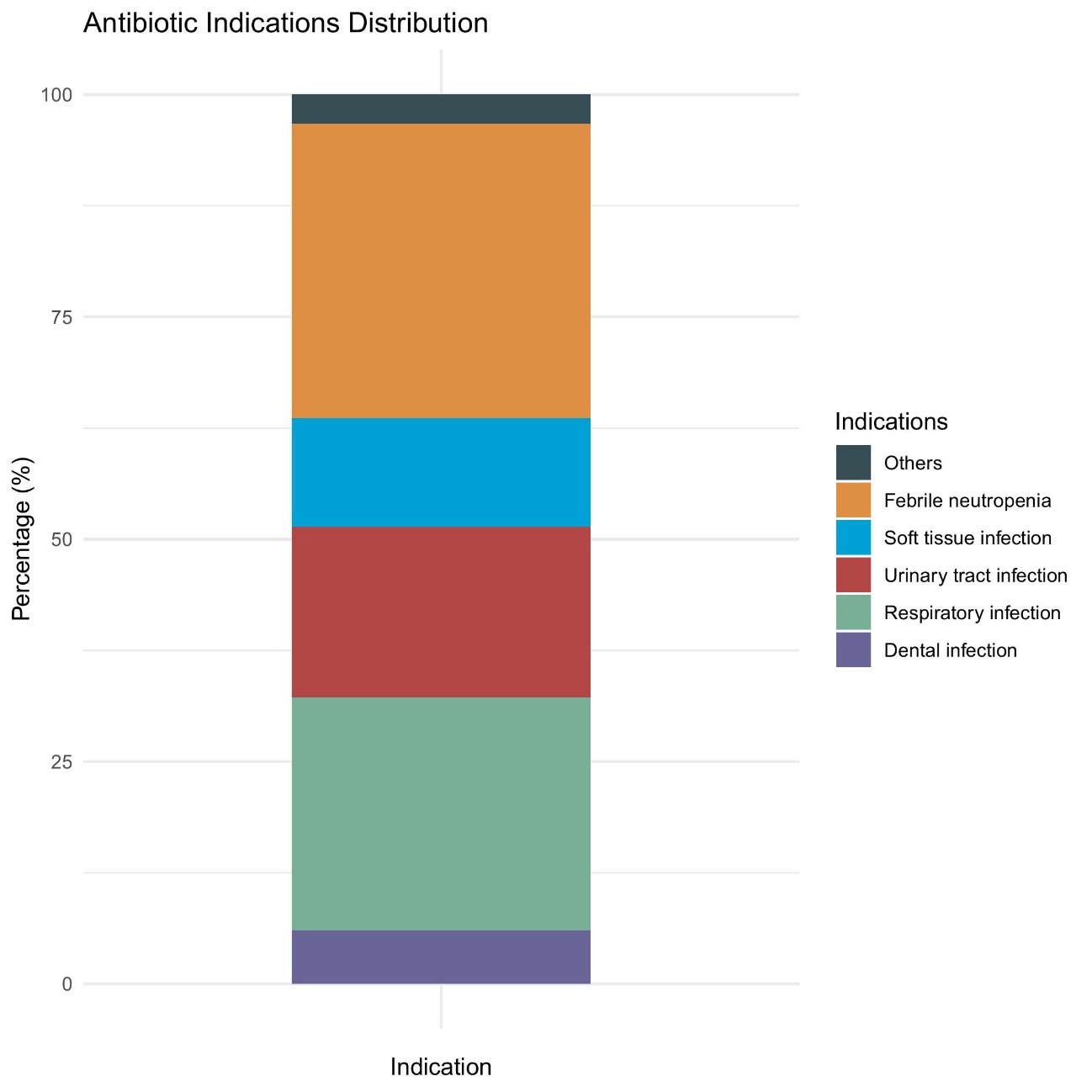


Distribution of clinical indications for antibiotic use. Percentages represent the relative frequency of each indication among antibiotic-exposed patients.

**
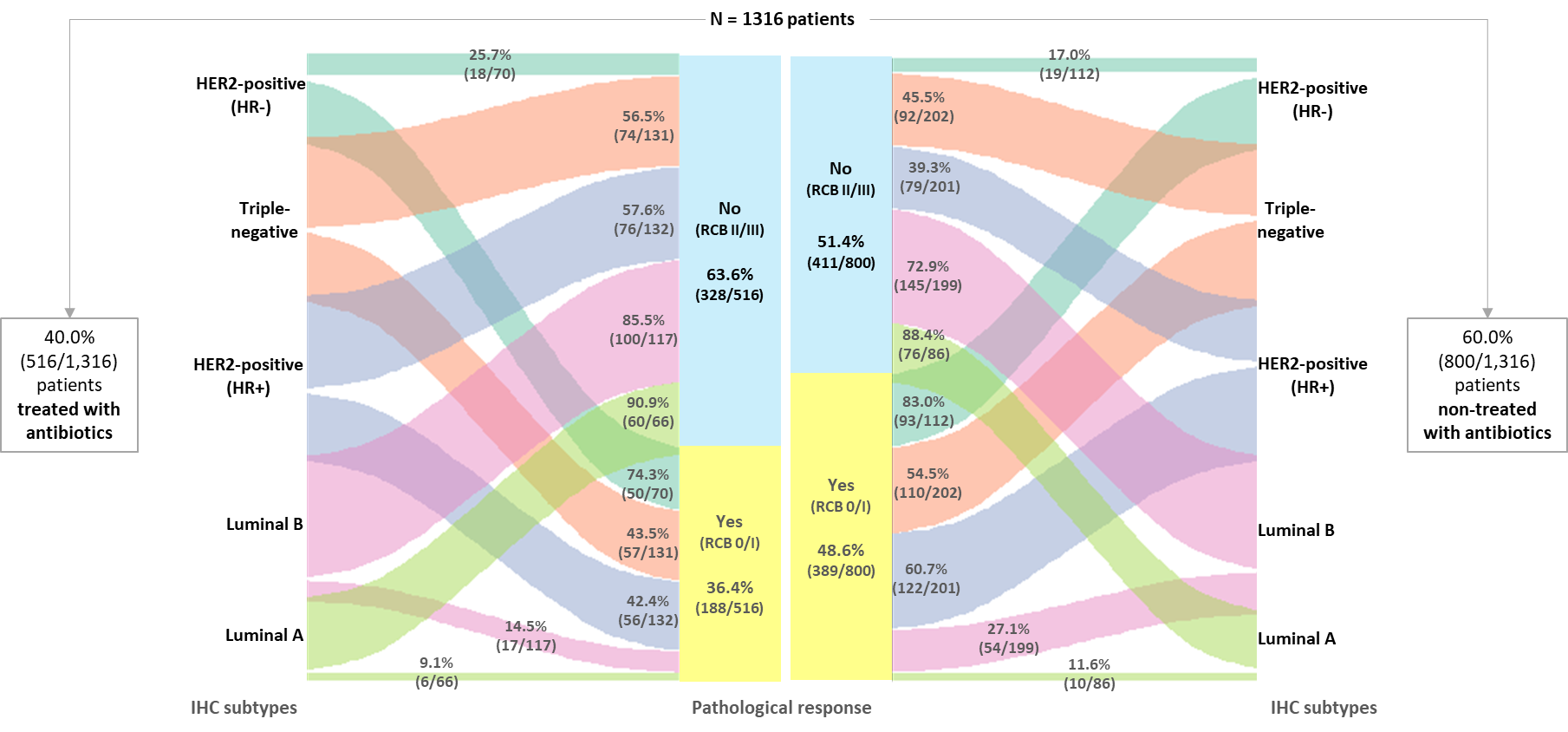
Figure S3.** **Distribution of Optimal Pathologic Response (RCB-0/I) by Immunohistochemical Subtype and Antibiotic Exposure**.

The Sankey diagram displays the observed distribution of patients by immunohistochemical subtype, antibiotic exposure, and optimal pathologic response. (RCB-0/I vs RCB-II/III). Antibiotic exposure was consistently associated with lower rates of optimal response across all subtypes. Statistical comparisons were conducted using 3 broad clinical subtypes and are reported in the Results section.

IHC, immunohistochemistry.

**
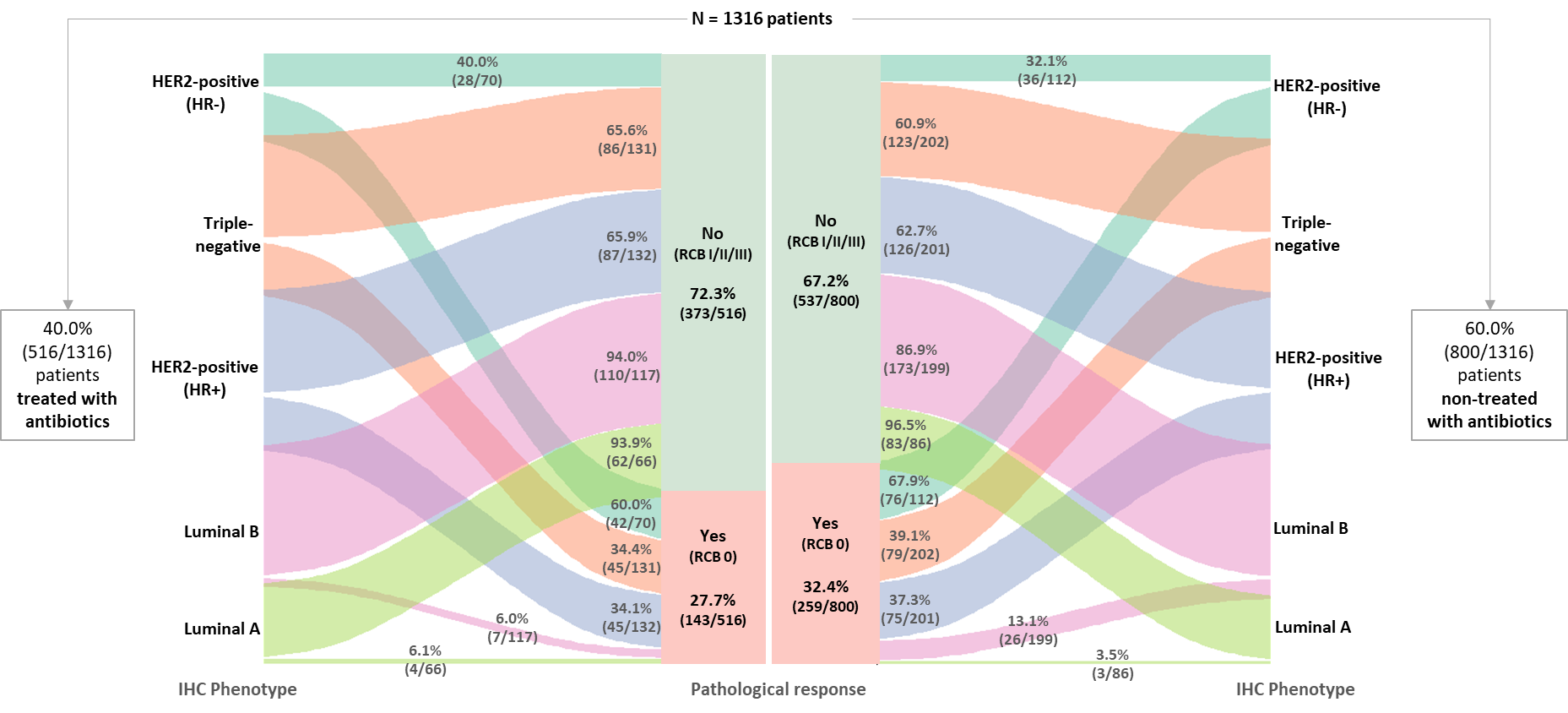
Figure S4. Distribution of Complete Pathologic Response (RCB-0) by Immunohistochemical Subtype and Antibiotic Exposure.**

The Sankey diagram displays the observed distribution of patients by immunohistochemical subtype, antibiotic exposure, and pathologic complete response (RCB-0 vs RCB-I/II/III). A consistent trend toward lower complete response rates was observed among patients exposed to antibiotics. Statistical comparisons were conducted using 3 broad clinical subtypes and are reported in the Results section.

IHC, immunohistochemistry.
