# Supplementary material for: Association between antibiotic use and pathologic response to neoadjuvant chemotherapy in breast cancer: a multicentre retrospective cohort study
Source: Breast. 2026 Jun 11;88:104833. doi: 10.1016/j.breast.2026.104833 (PMC13280407; doi:10.1016/j.breast.2026.104833)
Supplement: Multimedia component 3 [file mmc3.docx]

**Figure S1. Patient selection flowchart.**

Among 1870 patients with early breast cancer treated with neoadjuvant chemotherapy (January 2009–January 2024), 554 were excluded (lost to follow-up before surgery, n=194; immune checkpoint inhibitor–based regimens, n=28; incomplete data, n=332), yielding a final analytic cohort of 1,316 patients.

BC, breast cancer; ICI, immune checkpoint inhibitor; NACT, neoadjuvant chemotherapy.

**Figure S2. Clinical indications for antibiotic use.**

Distribution of clinical indications among antibiotic-exposed patients; percentages are calculated within the exposed group.

**Figure S3. Optimal pathologic response (RCB-0/I) by immunohistochemical subtype and antibiotic exposure.**

The Sankey diagram displays the observed distribution of patients by immunohistochemical subtype, antibiotic exposure, and optimal pathologic response. (RCB-0/I vs RCB-II/III). Antibiotic exposure was consistently associated with lower rates of optimal response across all subtypes. Statistical comparisons were conducted using 3 broad clinical subtypes and are reported in the Results section.

IHC, immunohistochemistry.

**Figure S4. Pathologic complete response (RCB-0) by immunohistochemical subtype and antibiotic exposure.**

The Sankey diagram displays the observed distribution of patients by immunohistochemical subtype, antibiotic exposure, and pathologic complete response (RCB-0 vs RCB-I/II/III). A consistent trend toward lower complete response rates was observed among patients exposed to antibiotics. Statistical comparisons were conducted using 3 broad clinical subtypes and are reported in the Results section.

IHC, immunohistochemistry.
